# The identification of established modifiable mid-life risk factors for cardiovascular disease which contribute to cognitive decline: Korean Longitudinal Study of Aging (KLoSA)

**DOI:** 10.1007/s40520-020-01783-x

**Published:** 2021-02-04

**Authors:** Yebeen Ysabelle Boo, Otto-Emil Jutila, Meghan A. Cupp, Logan Manikam, Sung-Il Cho

**Affiliations:** 1 Nuffield Department of Population Health, University of Oxford, Oxford, UK; 2 Population, Policy and Practice, UCL Great Ormond Street Institute of Child Health, London, UK; 3 Department of Epidemiology and Public Health, UCL Institute of Epidemiology and Health Care, London, UK; 4 Aceso Global Health Consultants Ltd, London, UK; 5 Brown University School of Public Health, Providence, Rhode Island, USA; 6 Graduate School of Public Health and Institute of Health and Environment, Seoul National University, Seoul, Republic of Korea

**Keywords:** Dementia, Mild cognitive impairment, Cardiovascular diseases, Korean longitudinal study of aging, Epidemiology

## Abstract

**Introduction:**

We explored how different chronic diseases, risk factors, and protective factors highly associated with cardiovascular diseases (CVD) are associated with dementia or Mild Cognitive Impairment (MCI) in Korean elders, with a focus on those that manifest in mid-life.

**Methods:**

A CVD-free cohort (*n* = 4289) from the Korean Longitudinal Study of Aging was selected to perform Cox mixed-effects proportional hazard regressions. Eighteen control variables with strong associations to CVD were chosen as explanatory variables, and Mini-Mental State Examination (MMSE) score cut-off for dementia and MCI were used as outcome variables.

**Results:**

The statistically significant (*P* < 0.05) adverse factors that contribute in developing dementia were age (aHR 1.07, 1.05–1.09), Centre for Epidemiological Studies Depression Scale (CESD-10) (aHR 1.17, 1.12–1.23), diagnosis with cerebrovascular disease (aHR 3.73, 1.81–7.66), living with diabetes (aHR 2.30, 1.22–4.35), and living with high blood pressure (HBP) (aHR 2.05, 1.09–3.87). In contrast, the statistically significant protective factors against developing dementia were current alcohol consumption (aHR 0.67, 0.46–0.99), higher educational attainment (aHR 0.36, 0.26–0.56), and regular exercise (aHR 0.37, 0.26–0.51). The factors with a statistically significant adverse association with progression to MCI were age (aHR 1.02, 1.01–1.03) and CESD-10 (aHR 1.17, 1.14–1.19). In contrast, the statistically significant protective factors against developing MCI were BMI (aHR 0.96, 0.94–0.98), higher educational attainment (aHR 0.33, 0.26–0.43), and regular exercise (aHR 0.83, 0.74–0.92).

**Conclusion:**

In lieu of the protective factor of MCI and dementia, implementing regular exercise routine well before mid-life and cognitive decline is significant, with adjustments made for those suffering from health conditions, so they can continue exercising despite their morbidity. Further attention in diabetes care and management is needed for patients who already show decline in cognitive ability as it is likely that their MCI impacts their ability to manage their existing chronic conditions, which may adversely affect their cognitive ability furthermore.

## Introduction

The 2016 Global Health Observatory data indicates that four of the top five causes of mortality worldwide are non-communicable diseases (NCDs) [1]. These NCDs include ischaemic heart disease, stroke, chronic obstructive pulmonary disease (COPD), and Alzheimer’s disease (AD) and other dementias. All these high burden NCDs are either a type of cardiovascular disease (CVD) or are closely associated with CVDs. This is indicative of the fact that CVD is the number one cause of mortality globally, with the World Health Organisation estimating mortality due to CVD at 17.9 million cases every year [2]. CVDs are a group of heart and blood vessel disorders, including coronary heart disease (CHD), cerebrovascular disease, and stroke. CVDs are an important comorbidity of COPD, as lower oxygen levels due to COPD may put additional strain on the heart and can contribute to heart failure [3]. Some well-understood non-modifiable risk factors for CVDs are age, ethnic background, and family history of CVDs. Modifiable risk factors include smoking, high blood pressure, diabetes, physical inactivity, being overweight or obese, and having high blood cholesterol [4]. Stress is also considered a risk factor for CVD, as high stress levels can either contribute to or be a consequence of a higher blood pressure, or an unhealthy lifestyle, such as habitual smoking, and increased alcohol consumption. There is also risk of reverse causality between socio-economic factors and health [5–7]. Socio-economic position is associated with an increased risk of developing NCDs, due to the social gradient in health and their life course epidemiology [8–10].

The mortality due to AD and other dementias, which more than doubled between 2000 and 2006, is one of the biggest challenges faced by the aging population [11]. It poses a major global public health crisis with great social, health, and economic costs, and the number of cases is predicted to double every 20 years [12]. The full extent of support and resources required by dementia patients is not captured, such as the impact on the quality of life for the caregiver and dementia patient. Dementia is an umbrella term to describe a group of neurodegenerative diseases which cause progressive and irreversible deterioration in cognitive function. Cognitive decline occurs in one or more cognitive domains: memory, emotion, behaviour, personality, visuospatial skills, and language [13]. The cognitive decline in dementia cases is severe enough to prevent the individual from functioning independently in daily or social activities [14]. There are several forms of dementia, including AD, vascular dementia, dementia with Lewy bodies, and Parkinson’s disease dementia. It is possible to simultaneously present with two or more forms of dementia, further complicating the classification and diagnosis of disease at autopsy [15]. Mild cognitive impairment (MCI) is often one of the first observable signs in the development of dementia, making it an important prognostic marker [16]. Patients with MCI have a greater cognitive decline compared to controls [17]. MCI primarily differs from dementia, since it is not severe enough to prevent the individual from undertaking complex activities [18]. Currently, it is estimated that between 10 and 20% of the world’s elderly population (aged 65 and above) is affected by MCI [19] and, although not all MCI patients will progress to further cognitive impairment or dementia, many MCI patients are identified to have a very mild form of dementia [20].

To date, there have been several longitudinal studies investigating the associations between cardiovascular risk factors and dementia [21–23]. Dementia, unlike other major chronic non-communicable diseases, has only symptomatic treatment; therefore, prevention by early detection and intervention is currently proposed to be the most effective approach for dementia and cognitive impairment [24–28]. Evidence from systematic reviews suggests that more than a third of all dementia cases could be prevented by addressing modifiable risk factors, with CVD (e.g., high blood pressure, HBP) and CVD-related risk factors (e.g., smoking) being significant influences [29]. Observing these risks that manifest in mid-life is particularly significant, as it is a vital point when exposure to risk factors may distinguish between normal aging and the development of dementia [30]. Interventions targeted at the appropriate modifiable risk factors during mid-life may be the most effective in preventing AD; however, this exact process and aetiological element remains unknown [13, 31].

This study focuses on the Republic of Korea (South Korea) population using Korean Longitudinal Study of Aging (KLoSA) data. Associations between modifiable CVD mid-life risk factors and cognitive decline have not been explored in great depth through KLoSA, despite South Korea having one of the most rapidly aging populations and a greater prevalence of dementia than other Asian and Western countries [32]. In fact, these Korean population studies investigating cognition function often focus on a singular factor [33–35]. To further explore risk for cognitive decline and dementia, this study utilises data from five waves of the KLoSA to explore how different chronic diseases and risk factors affect the Mini-Mental State Exam (MMSE) score from 2006 to 2016. This study is distinct from prior work, since the Korean population is rarely a main ethnic group in other well-established longitudinal studies of modifiable risk factors and cognitive health from western populations. This is significant since, even with increasing westernisation, Koreans may have specific environmental, social, lifestyle, and genetic factors, which contribute to dementia and CVD risk [36–40]. In particular, Asian individuals experience greater risk of diabetes and HBP at a lower BMI, compared to White counterparts [41] and have a high prevalence of novel genetic variations related to salt sensitivity which can increase the risk of HBP and other CVD events [42, 43]. For example, Koreans have a lower cut-off points for BMI-defined obesity, compared to White individuals, due to having an elevated body fat levels at the same BMI and increased risk of obesity-related cardiometabolic diseases [56]. Therefore, to the best of our knowledge, this is the first completed longitudinal countrywide study involving a middle-age population-based cohort study for investigating the association of CVD, CVD risk factors, and lifestyle factors, with all-cause MCI and dementia in a Korean population. This study, which utilises data from an under-researched ethnic group, will allow for the comparison of associations found from literature using international data and will add to existing knowledge about targeted health interventions to improve the health and well-being of aging populations.

## Methods

**Fig. 1:**
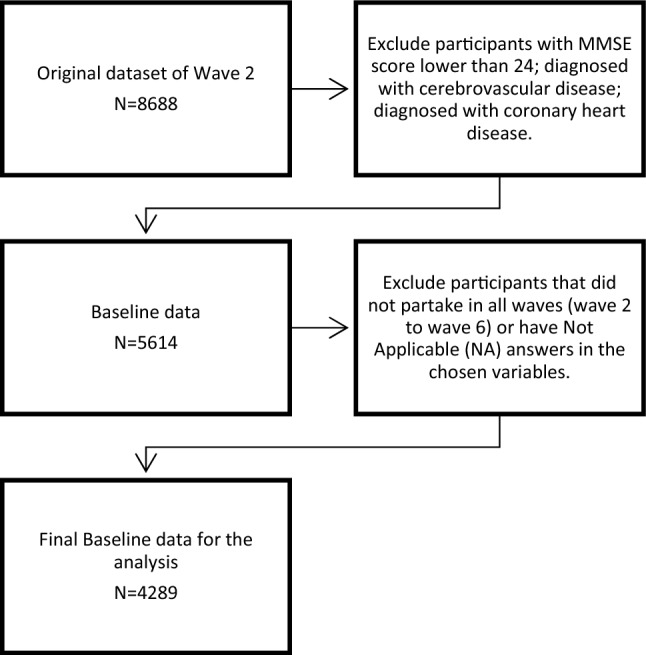
Flowchart for participant data selection and inclusion and exclusion criteria for study

The KLoSA dataset includes a random sample of 10,254 adults born before 1961, who are 45 or older at the time of data collection and reside in Korea (excluding Jeju Island). Initiated in 2006, the baseline survey is carried out every two years and six waves are completed as of 2020. A refreshment sample of 920 individuals born between 1962 and 1963 was added to wave 5 in 2014 as before this, KLoSA only hold a closed cohort. This study only utilises data collected from participants of wave 2 and subsequent waves, as institutionalised individuals (e.g., those in assisting living) were initially excluded from wave 1. More information about KLoSA design can be found in the Division of Behavioral and Social Research (DBSR) International Studies Behavioral and Social Studies DBSR Korean Longitudinal Study of Aging website [44]. Figure 1 shows the criteria which were applied to exclude participants from the original dataset of wave 2. The final baseline dataset used for data analysis contained 4,289 participants. The participants previously diagnosed with CHD and cerebrovascular diseases were excluded to allow for the assessment of how the development of new chronic diseases or existing lifestyle risk factors affect cognitive decline over time. Twenty-one percent of participants had one or more missing answers. However, out of all variables in the dataset, only nine variables had partial missing data. We excluded participants using list-wise deletion if they had not applicable (NA) answers during wave 2 collection point, or if they did not partake in all waves (2–6) [45].

### Variable selection

A total of 16 control variables were chosen to perform Cox mixed-effects proportional hazard regression models with MMSE score as the outcome variable. The MMSE, developed by Folstein in 1975 [46], is a screening test that measures cognitive ability and is often used as a diagnostic tool for MCI. The test is delivered in 7–10 min, and is designed to measure the general cognitive ability of orientation, registration, attention-calculation, recall, and language. It is composed of 30 question scores between 18 and 23 indicate cognitive deficiency and scores 17 and below indicates dementia [47]. The MMSE has a sensitivity of 86.36% and specificity of 86.36% for patients who have been educated for five years or more [48]. Multiple measurements of MMSE changes over time can be used to predict the conversion of MCI to dementia [49]. There are few limitations with MMSE itself as a measurement of cognitive skills. It does not account for individual differences; people who have low educational level or in low socio-economic status, they score relatively low due to floor effect (low specificity), whereas with highly educated people, they score relatively high due to celling effect (low sensitivity).

The following independent variables were chosen to reflect both the physical and mental health of the participants and lifestyle and socio-economic determinates of health:

1. Two mental health components: 10-item revised Centre for Epidemiological Studies Depression Scale (CESD-10); diagnosed with mental health disability;
2. Lifestyle risk factors: Smokes cigarette, drinking habits, Body Mass Index (BMI), regular exercise;
3. Pre-existing conditions: Diagnosed with arthritis or rheumatism; liver diseases; lung diseases; cancer; diabetes; HBP;
4. Newly diagnosed with CVD: cerebrovascular diseases; CHD;
5. Highest year of education.

These independent factors can be sub-grouped as protective factors and risk factors. Protective factors include regular exercise, low CESD-10 score, high satisfaction with economics status, and higher level of education. The risk factors include being diagnosed with various chronic diseases, smoking, and/or drinking habits and high BMI. The biological explanation linking the risk factors of CVD with the risk of cognitive decline provides the rationale of selecting these variables. For example, HBP is a well-known modifiable CVD risk factor related to impairment in cognitive function and vascular dementia, characterised by alterations in the cerebral vasculature, function, and blood flow [29].

Smoking is another independent risk for CVD, and there is a consensus that it can increase the risk of dementia and cognitive impairment. Smoking is part of multiple pathological pathways for cognitive decline, and is an independent risk factor for stroke, atherosclerosis, inflammatory processes, and oxidative damage to neurons [53]. Moreover, type-II diabetes mellitus (T2DM) is firmly linked with negative alterations to cognitive function, through several domains. Several mechanisms both vascular and non-vascular (hormonal) are proposed to contribute to cognitive decline and the pathophysiological process for T2DM and dementia have a significant overlap [54].

Another example is obesity, which has rapidly increased in the last couple of decades in Korea [55]. A plausible link between obesity and cognition occurs through an indirect effect on CVD risk factors [57]. Finally, while the relationship between alcohol and cognitive decline and dementia has not been firmly established, low-to-moderate alcohol consumption has been suggested to be cardio-protective and even neuroprotective [62], albeit, heavy consumption is neurotoxic and has been associated with a higher risk for cognitive impairment and dementia in later life [63]. However, not many studies have explored the role of ethnicity or Korean population in alcohol consumption and development of dementia.

In contrast, following three non-modifiable variables were included in the model to control any biological or time-varying factors:

1. Wave of the interview (every two years from 2006 up to 2016)
2. Age
3. Gender
4. Respondent’s satisfaction with economic status (as a proxy for subjective wealth due to different living cost being associated with different regions of Korea).

### Statistical analysis

The Chi-squared test and Kruskal-Wallis one-way analysis of variance test were utilised for analysis of categorical and continuous variables, respectively. These analyses conducted to see the initial correlation of each variable against the MMSE score, via descriptive analysis. The univariate Cox proportional hazard regression models were conducted for six modifiable factors and were visualised as graphical plots. The included six modifiable factors were:

1. CESD-10 *(0* = *lowest to 10* = *highest score)*
2. smoking *(0* = *non-smoker, 1* = *past smoker, 2* = *current smoker)*
3. alcohol consumption *(0* = *non-drinker, 1* = *past drinker, 2* = *current drinker)*
4. HBP which can also reflect stress, as well as respondent’s underlying conditions (0 = no, 1 = yes)
5. total number of chronic diseases (*sumchron, Min* = *0, Max* = *6)*
6. *regular exercise* (0 = no, 1 = yes).

Cox mixed-effects proportional hazard regression models were also used to confirm how each factor influenced the development of dementia (model 1 with MMSE score ≤ 17) and MCI (model 2 with MMSE score 18–23), controlling the random effects of each patient. The rationale behind building two models, with different cut-off points, is to observe and compare which variables accelerate cognitive decline, while the participants show normal cognitive scores relative to the point when they already have a MCI condition (sensitivity analysis). Cox regression models are also useful in assessing survival time, which is appropriate for our data. Model 1 will show which variables accelerate the event of cognitive decline to dementia, while model 2 will show which variables influence the event of cognitive decline up to the development of MCI. The division of two models will allow for the investigation of the effect modifiable risk factors for both MCI and dementia.

## Results

A descriptive analysis of the participants at the beginning of this study in relation to each MMSE score (1–30) can be found in Table 1.

**Table 1:** Descriptive analysis of the participants (*n* = 4289)

MMSE score	24 (*N* = 275)	25 (*N* = 352)	26 (*N* = 466)	27 (*N* = 560)	28 (*N* = 704)	29 (*N* = 835)	30 (*N* = 1097)	Total (*N* = 4289)	*p* value
CESD-10									< 0.001
Mean (SD)	4.07 (2.90)	3.53 (2.67)	3.50 (2.69)	3.33 (2.63)	3.00 (2.54)	2.58 (2.43)	2.34 (2.47)	2.96 (2.62)	
Median (Q1, Q3)	4.00 (1.00, 7.00)	3.00 (2.00, 6.00)	3.00 (1.00, 6.00)	3.00 (1.00, 5.00)	2.00 (1.00, 5.00)	2.00 (0.00, 4.00)	2.00 (0.00, 4.00)	2.00 (1.00, 5.00)	
Min–Max	0.00–10.00	0.00–10.00	0.00–10.00	0.00–10.00	0.00–10.00	0.00–10.00	0.00–10.00	0.00–10.00	
Alcohol									0.051
Non-drinker	149 (54.2%)	193 (54.8%)	225 (48.3%)	289 (51.6%)	326 (46.3%)	390 (46.7%)	519 (47.3%)	2091 (48.8%)	
Past drinker	21 (7.6%)	29 (8.2%)	27 (5.8%)	39 (7.0%)	56 (8.0%)	61 (7.3%)	69 (6.3%)	302 (7.0%)	
Current drinker	105 (38.2%)	130 (36.9%)	214 (45.9%)	232 (41.4%)	322 (45.7%)	384 (46.0%)	509 (46.4%)	1896 (44.2%)	
Smoking									0.042
Non-smoker	183 (66.5%)	259 (73.6%)	328 (70.4%)	385 (68.8%)	466 (66.2%)	548 (65.6%)	730 (66.5%)	2899 (67.6%)	
Past smoker	28 (10.2%)	45 (12.8%)	45 (9.7%)	76 (13.6%)	89 (12.6%)	109 (13.1%)	127 (11.6%)	519 (12.1%)	
Current smoker	64 (23.3%)	48 (13.6%)	93 (20.0%)	99 (17.7%)	149 (21.2%)	178 (21.3%)	240 (21.9%)	871 (20.3%)	
BMI									0.011
Mean (SD)	23.17 (2.89)	23.09 (2.59)	23.49 (2.60)	23.13 (2.57)	23.43 (2.52)	23.50 (2.43)	23.25 (2.37)	23.32 (2.51)	
Median (Q1, Q3)	22.89 (21.45, 25.00)	22.86 (21.46, 24.63)	23.48 (21.78, 24.97)	23.17 (21.35, 24.61)	23.31 (21.64, 24.97)	23.39 (21.94, 24.91)	23.18 (21.72, 24.54)	23.23 (21.64, 24.80)	
Min–Max	15.40–36.11	16.00–31.64	17.26–33.98	13.15–32.89	16.82–31.63	16.69–31.53	16.33–34.48	13.15–36.11	
Arthritis or rheumatism									< 0.001
No	208 (75.6%)	284 (80.7%)	352 (75.5%)	480 (85.7%)	584 (83.0%)	725 (86.8%)	995 (90.7%)	3628 (84.6%)	
Yes	67 (24.4%)	68 (19.3%)	114 (24.5%)	80 (14.3%)	120 (17.0%)	110 (13.2%)	102 (9.3%)	661 (15.4%)	
Mental illness									0.376
No	272 (98.9%)	344 (97.7%)	462 (99.1%)	552 (98.6%)	695 (98.7%)	821 (98.3%)	1088 (99.2%)	4234 (98.7%)	
Yes	3 (1.1%)	8 (2.3%)	4 (0.9%)	8 (1.4%)	9 (1.3%)	14 (1.7%)	9 (0.8%)	55 (1.3%)	
Liver diseases									0.135
No	269 (97.8%)	346 (98.3%)	463 (99.4%)	547 (97.7%)	687 (97.6%)	818 (98.0%)	1085 (98.9%)	4215 (98.3%)	
Yes	6 (2.2%)	6 (1.7%)	3 (0.6%)	13 (2.3%)	17 (2.4%)	17 (2.0%)	12 (1.1%)	74 (1.7%)	
Lung diseases									0.15
No	269 (97.8%)	342 (97.2%)	453 (97.2%)	548 (97.9%)	696 (98.9%)	818 (98.0%)	1084 (98.8%)	4210 (98.2%)	
Yes	6 (2.2%)	10 (2.8%)	13 (2.8%)	12 (2.1%)	8 (1.1%)	17 (2.0%)	13 (1.2%)	79 (1.8%)	
Cancer									0.487
No	267 (97.1%)	338 (96.0%)	456 (97.9%)	540 (96.4%)	687 (97.6%)	816 (97.7%)	1070 (97.5%)	4174 (97.3%)	
Yes	8 (2.9%)	14 (4.0%)	10 (2.1%)	20 (3.6%)	17 (2.4%)	19 (2.3%)	27 (2.5%)	115 (2.7%)	
Diabetes									< 0.001
No	232 (84.4%)	297 (84.4%)	404 (86.7%)	503 (89.8%)	640 (90.9%)	750 (89.8%)	1016 (92.6%)	3842 (89.6%)	
Yes	43 (15.6%)	55 (15.6%)	62 (13.3%)	57 (10.2%)	64 (9.1%)	85 (10.2%)	81 (7.4%)	447 (10.4%)	
High blood pressure									< 0.001
No	185 (67.3%)	257 (73.0%)	316 (67.8%)	430 (76.8%)	516 (73.3%)	622 (74.5%)	895 (81.6%)	3221 (75.1%)	
Yes	90 (32.7%)	95 (27.0%)	150 (32.2%)	130 (23.2%)	188 (26.7%)	213 (25.5%)	202 (18.4%)	1068 (24.9%)	
Total number of chronic diseases									< 0.001
Mean (SD)	0.85 (0.93)	0.75 (0.86)	0.79 (0.90)	0.61 (0.81)	0.63 (0.80)	0.61 (0.81)	0.43 (0.70)	0.62 (0.82)	
Median (Q1, Q3)	1.00 (0.00, 1.00)	1.00 (0.00, 1.00)	1.00 (0.00, 1.00)	0.00 (0.00, 1.00)	0.00 (0.00, 1.00)	0.00 (0.00, 1.00)	0.00 (0.00, 1.00)	0.00 (0.00, 1.00)	
Min–Max	0.00–4.00	0.00–4.00	0.00–4.00	0.00–4.00	0.00–4.00	0.00–4.00	0.00–4.00	0.00–4.00	
Wealth satisfaction									< 0.001
Mean (SD)	49.71 (21.44)	50.82 (20.91)	51.82 (20.80)	54.04 (20.30)	53.81 (21.61)	56.71 (20.54)	57.47 (19.72)	54.61 (20.75)	
Median (Q1, Q3)	50.00 (40.00, 70.00)	50.00 (40.00, 70.00)	50.00 (40.00, 70.00)	50.00 (40.00, 70.00)	50.00 (40.00, 70.00)	60.00 (45.00, 70.00)	60.00 (50.00, 70.00)	50.00 (40.00, 70.00)	
Min–Max	0.00–100.00	0.00–100.00	0.00–100.00	0.00–100.00	0.00–100.00	0.00–100.00	0.00–100.00	0.00–100.00	
Educational attainment									< 0.001
Elementary	166 (60.4%)	179 (50.9%)	219 (47.0%)	199 (35.5%)	251 (35.7%)	216 (25.9%)	181 (16.5%)	1411 (32.9%)	
Middle	44 (16.0%)	62 (17.6%)	95 (20.4%)	128 (22.9%)	155 (22.0%)	168 (20.1%)	206 (18.8%)	858 (20.0%)	
High	57 (20.7%)	83 (23.6%)	119 (25.5%)	183 (32.7%)	222 (31.5%)	329 (39.4%)	492 (44.8%)	1485 (34.6%)	
College/University	8 (2.9%)	28 (8.0%)	33 (7.1%)	50 (8.9%)	76 (10.8%)	122 (14.6%)	218 (19.9%)	535 (12.5%)	
Gender									< 0.001
Male	122 (44.4%)	137 (38.9%)	201 (43.1%)	262 (46.8%)	331 (47.0%)	425 (50.9%)	559 (51.0%)	2037 (47.5%)	
Female	153 (55.6%)	215 (61.1%)	265 (56.9%)	298 (53.2%)	373 (53.0%)	410 (49.1%)	538 (49.0%)	2252 (52.5%)	
Age									< 0.001
Mean (SD)	64.16 (8.84)	62.68 (9.15)	61.28 (8.72)	60.09 (8.92)	59.70 (8.69)	58.68 (8.31)	56.52 (7.40)	59.44 (8.68)	
Median (Q1, Q3)	64.00 (57.00, 71.00)	63.00 (55.75, 69.00)	61.00 (54.00, 68.00)	59.00 (53.00, 67.00)	59.00 (52.00, 66.00)	58.00 (52.00, 64.50)	55.00 (50.00, 61.00)	58.00 (52.00, 66.00)	
Min–Max	47.00–91.00	47.00–87.00	47.00–84.00	47.00–87.00	47.00–85.00	47.00–88.00	47.00–81.00	47.00–91.00	
Regular exercise									< 0.001
No	185 (67.3%)	220 (62.5%)	305 (65.5%)	337 (60.2%)	397 (56.4%)	480 (57.5%)	573 (52.2%)	2497 (58.2%)	
Yes	90 (32.7%)	132 (37.5%)	161 (34.5%)	223 (39.8%)	307 (43.6%)	355 (42.5%)	524 (47.8%)	1792 (41.8%)	

10 variables out of 17 showed statistical significance when tested for correlation with MMSE score. These variables were *CESD-10*, presence of joint conditions; *diabetes*; *HBP* conditions, total number of chronic diseases (*sumchron*), *wealth* satisfaction, highest *education* level, *gender, age*, and *regular exercise* behaviors

**Fig. 2:**
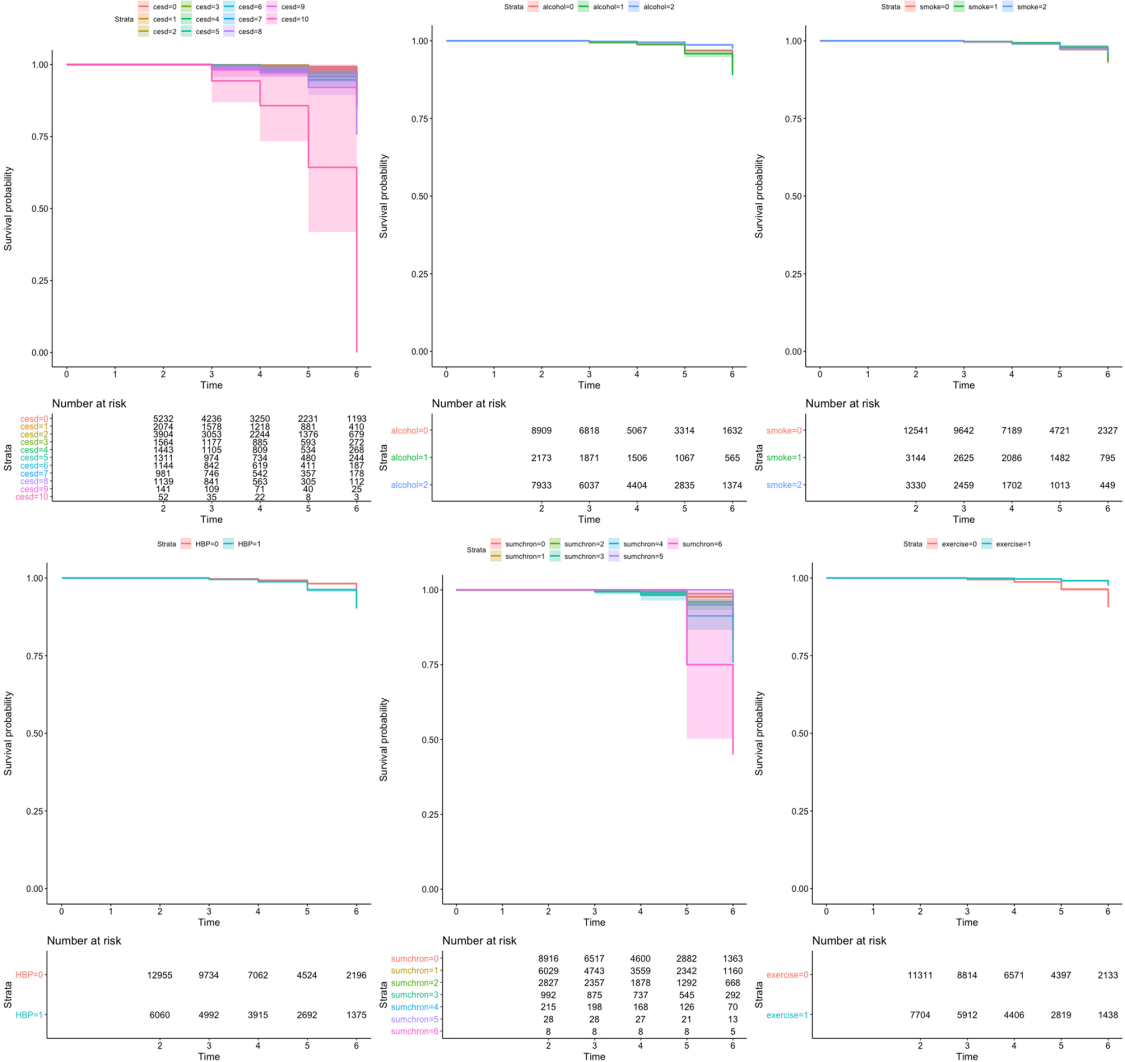
Model 1: the univariate Cox proportional hazard regression models for six modifiable factors using MMSE score cut-off for dementia (≤ 17) as a hazard outcome, five cumulative waves (2–6)

**Fig. 3:**
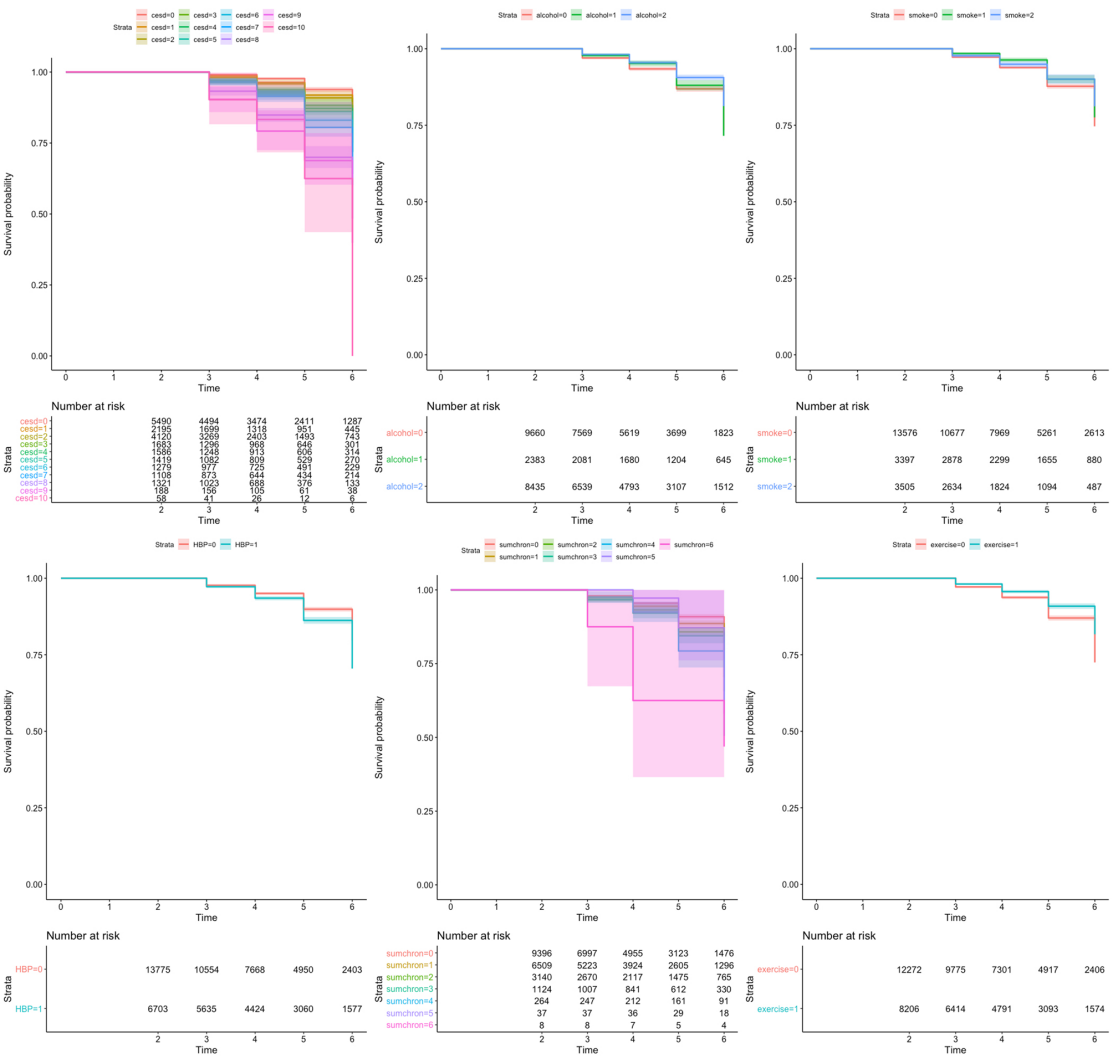
Model 2: the univariate Cox proportional hazard regression models for six modifiable factors using MMSE score cut-off for MCI (18 ≤ x ≤ 23) as a hazard outcome, five cumulative waves (2–6)

Figures 2 and 3 display the univariate Cox proportional hazard regression models for six selected modifiable factors. The drinking and smoking status of the respondents (which were not statistically significant in Chi-square test) did not have a significant protective effect on the event of cognitive decline over time (for model 1 or 2). However, high scores on *CESD-10*, higher total number of chronic diseases *(sumchron*), presence of *HBP*, and respondents without *regular exercise* showed risks of developing both dementia and MCI.

Table 2 represents adjusted hazard ratios (aHR) of Cox mixed-effects proportional hazard regression of dementia (model 1) and MCI (model 2). Model 1 uses maximum-likelihood ratio tests based on the integrated and penalized views of the model, along with penalized values. The hazard ratios presented are the results after controlling individual effects as well as other co-variates. A variance of 1.81 (with standard deviation of 1.34) was observed in model 1, while a variance of 0.65 (with standard deviation of 0.80) was observed in model 2.

**Table 2:** Adjusted hazard ratios of Cox mixed-effects proportional hazard regression of dementia (model 1) and MCI (model 2)

	Model 1: hazard ratio (adjusted)	Model 2: hazard ratio (adjusted)
Gender (ref: male)		
Female	1.26 (0.82–1.93)	1.10 (0.92–1.31)
Age	1.07*** (1.05–1.09)	1.02*** (1.01–1.03)
BMI (kg/m^2^)	0.96 (0.92–1.01)	0.96*** (0.94–0.98)
CESD-10 (Min:0, Max:10)	1.17*** (1.12–1.23)	1.17*** (1.14–1.19)
Alcohol (ref: non-drinker)		
Past drinker	1.26 (0.87–1.84)	0.96 (0.81–1.14)
Current drinker	0.67** (0.46–0.99)	0.94 (0.81–1.08)
Smoke (ref: non-smoker)		
Past smoker	1.08 (0.69–1.70)	0.94 (0.78–1.14)
Current smoker	1.42 (0.86–2.34)	0.96 (0.78–1.18)
Newly diagnosed with cardiovascular diseases (ref: never diagnosed)		
Coronary heart disease	1.30 (0.56–3.02)	1.00 (0.71–1.41)
Cerebrovascular disease	3.73*** (1.81–7.66)	1.00 (0.68–1.47)
Living with chronic illness (ref: absent)		
Arthritis or rheumatism	1.81* (0.95–3.43)	1.06 (0.81–1.38)
Mental illness	1.42 (0.62–3.29)	1.03 (0.71–1.48)
Liver diseases	0.73 (0.22–2.43)	1.06 (0.71–1.59)
Lung diseases	1.53 (0.61–3.85)	0.95 (0.64–1.39)
Cancer	1.81 (0.84–3.90)	0.79 (0.57–1.10)
Diabetes	2.30** (1.22–4.35)	0.94 (0.72–1.23)
High blood pressure	2.05** (1.09–3.87)	1.13 (0.88–1.47)
Wealth satisfaction (Min:0, Max:10)	0.98*** (0.98–0.99)	0.99*** (0.99–0.99)
Educational attainment (ref: Elementary school)		
Middle school	0.46*** (0.31–0.69)	0.64*** (0.55–0.74)
High school	0.38*** (0.26–0.56)	0.42*** (0.36–0.48)
College/university	0.40*** (0.21–0.75)	0.33*** (0.26–0.43)
Regular exercise (ref: absent)	0.37*** (0.26–0.51)	0.83*** (0.74–0.92)

***p < 0.01, **p < 0.05, *p < 0.1

In Model 1, the statistically significant (*P* < 0.05) adverse factors associated with the development of dementia were age (aHR 1.07, 1.05–1.09), CESD-10 (each unit increase accounts for aHR 1.17, 1.12–1.23), diagnosis with cerebrovascular disease (aHR 3.73, 1.81–7.66), living with diabetes (aHR 2.30, 1.22–4.35), and living with HBP (aHR 2.05, 1.09–3.87). In contrast, the statistically significant protective factors against developing dementia were current alcohol consumption (aHR 0.67, 0.46–0.99), increase in wealth satisfaction (each unit increase in satisfaction accounts for aHR 0.98, 0.98–0.99), higher educational attainment (aHR 0.36, 0.26–0.56), and regular exercise (aHR 0.37, 0.26–0.51).

In Model 2, the statistically significant adverse factors that affected the event of decline in cognitive ability to MCI were age (aHR 1.02, 1.01–1.03) and CESD-10 (each unit increase accounts for aHR 1.17, 1.14–1.19). In contrast, the statistically significant protective factors against developing MCI were BMI (every unit increase in kg/m^2^ accounts for aHR 0.96, 0.94–0.98), increase in wealth satisfaction (each unit increase in satisfaction accounts for aHR 0.99, 0.99–0.99), higher educational attainment (aHR 0.33, 0.26–0.43), and regular exercise (aHR 0.83, 0.74–0.92).

## Discussion

Using data from this large cohort representative of the Korea population, aged 45 or older who were free from pre-existing cerebrovascular disease and CHD, we observed several factors that contribute significantly in developing dementia (model 1). When adjusting for age, lifestyle factors, socio-economic determinates, and existing illnesses, being newly diagnosed with cerebrovascular disease increased the risk of developing dementia 3.73 times. While not statistically significant, being newly diagnosed with CHD increases the risk 1.3 times compared to those who do not. Almost all existing chronic diseases increased the risk of dementia (after adjustment), with existing diabetes condition showing the greatest statistically significant risk for developing dementia (2.3 times more than those who do not have diabetes). Existing HBP condition also showed great risks in developing dementia; those who have HBP were 2.05 times more likely to develop dementia. In addition, arthritis and rheumatism also increased the risks of developing dementia, which contrasts with the lifestyle factor of regular exercise—which may suggest reverse causality due to limited movement and joint pain leading to reduced ability to exercise routinely. Similar trends were shown in MCI (model 2), with some exceptions of liver diseases, lung diseases, cancer, and diabetes. Therefore, it was noted that while liver diseases may accelerate cognitive decline before the MCI (rather than after), lung diseases, cancer, and diabetes do not contribute to cognitive decline until the patients reach their MCI stage. Being newly diagnosed with CHD or cerebrovascular disease also shows effects only after MCI stage is reached, but not before.

This is in agreement with White and colleagues who found through post-mortem examinations a strong association between dementia and death from cerebrovascular disease [64]. The association between HBP and increased risk of dementia and MCI in later life is similar to a recent nationwide cohort study in Korea, in which they found a linear association between increased blood pressure and all-type dementia [65]. The association of diabetes with a higher risk in all-cause dementia also agrees with the majority of the literature and other Korean countrywide population studies [35], while the difference in the results between two models may be due to variation in diabetes management [66]. For instances, patients who already faced MCI may have lack of capacity in managing their diabetes compared to those who have not. While diabetes may not advance cognitive decline before MCI, if a diabetes management plan is well followed, the worsening of diabetic conditions due to MCI can accelerate further cognitive decline and contribute to dementia.

Interestingly, while each unit increase in severity of depressive symptoms (CESD-10) increased the risks of developing both MCI and dementia, when we controlled the depressive symptoms, both alcohol status and smoking status showed protective effects to MCI. However, it is important to consider that drinking and smoking variables had a three-level classification in this study—whilst there can often be a great difference in the level of consumption of alcohol or number of cigarettes smoked per week. As well, heavy drinkers and smokers often have a significantly shorter life expectancy and can die before the late-life manifestation of MCI [67, 68]. Moreover, the present study could demonstrate a pattern of lower dementia risk for past smokers when compared to current smokers which aligns with numerous studies [69, 70]. The lack of a significant difference in this study could be attributed to variation in the period of smoking cessation. Another longitudinal study by Choi and colleagues found a significant decrease risk for dementia in long-term past smokers' Korean men, however, not for short-term quitters [33]. The assessment of the smoking cessation period is required in future studies.

In contrast, in terms of risks of developing dementia, past drinkers showed higher risks than current drinkers. While the lack of association between current drinking and dementia could be due to differences in the volume of alcohol consumption, low to moderate levels of alcohol consumption has been suggested to have no effect [71] or even decreased risk for dementia and cognitive decline [72]. The potential neuroprotective effects could be attributed reducing the effects other of CVD risk factors and a healthy lifestyle pattern [73], and polyphenols in Korean rice wine [74, 75]. However, the negative effects of chronic high alcohol consumption may supersede any potential positive effects [76]. An extensive umbrella review found an association of excess alcohol consumption and dementia [77]. Moreover, former drinkers may have stopped their alcohol consumption due to the health consequences [78], and this could overestimate the benefits of continued alcohol use and increased risk when compared to those who never drink. oftentimes, individuals may cease drinking because of sickness that may be unobserved in the study, leaving former drinkers with higher risk. In addition, current drinkers with alcohol use disorders (AUD) are often underrepresented and have substantially shortened lifespan compared to former AUD individuals [79, 80]. A previous diagnosis of AUD has a greater risk for all-cause dementia than all other modifiable risk factors [77]. Therefore, the adverse effects of drinking heavily before the age of 45 may be reflected in their cognitive decline over time [81]. In addition, these results can reflect an institution's policy of no drinking or no smoking for the participants who are residing in institutions (i.e., care homes).

High BMI was also a protective factor in developing dementia and MCI, in agreement with previous research [82–85]. This mirrors the descriptive analysis and the literature which showed a low prevalence of obese and overweight Koreans born before 1970 [86]. The protective effect of a high BMI may be attributed reverse causality, since prodromal dementia and MCI are associated with weight loss [87]. In contrast, it is also important to note that higher BMI can reduce life expectancy and individuals will pass away before the development of dementia [88]. Nevertheless, obesity’s association with cognitive impairment and dementia has been contradictory; while mid-life obesity has been identified as a risk factor [58], it was observed to be protective in later life [59]. In one Korean-based population study, late-life obesity was associated with dementia [60], while another study found no association between baseline BMI and dementia, except in underweight males [61]. The fundamental relationship between BMI and dementia is not conclusive in Korean populations. On the other hand, key limitations in using the BMI measurement for obesity is that it does not distinguish between lean muscle and fat, and underestimates adiposity in the elderly [89]. Further studies should consider supplementary measurements (skinfold thickness and waist circumstance) to better determine obesity status. Finally, both high educational and occupational attainment have been suggested to contribute to a “cognitive reserve” that can slow down cognitive decline related to AD and delay onset [90]. This effect was also observed in our study, having higher education, greater wealth satisfaction, and regular exercise behaviors showed statistically significant protective results in developing both dementia and MCI, most likely due to practicing healthy lifestyle as a result of having higher socio-economic positions and resources being available for them to practice such protective factors.

### Limitations

In this study, a list-wise deletion was used to handle the missing values and dropout before wave 6. Since the missing values are likely to be Missing-Not-At-Random (MNAR) due to the participants’ desire not to give out a sensitive information about themselves, it can underestimate the association between the risk factors to dementia and MCI. Also, the using list-wise deletion to treat missing data may have deleted the important data that may greatly influence the outcome variables (thus, creating a selection bias and attrition bias). However, out of whole dataset, only nine variables had missingness at wave 2 and each variable showed less than 7% of missingness. In addition, retention rate from wave 2 to 6 was 76%, which is above critical value (70%). Another limitation is that despite both participants’ surveys and health professionals’ opinion on medical/biological conditions used in KLoSA, the participants may suffer from recall bias or misinterpretation during the interview, which could have led to random misclassification and bias toward the null value.

A fundamental limitation is that this study only covers ten years, while often extended preclinical duration of dementia can take up to 20 years before diagnosis. It may be an insufficient period for symptoms to manifest from a mid-life population. Further continuation of this cohort study would overcome this limitation and ameliorate possible reverse causality. The MMSE test is also insufficient to diagnosis or exclude dementia entirely, and to differentiate between MCI and dementia. A study by Hensel and colleagues showed that the reliable change between wave 1–5 of the longitudinal study on the MMSE score is − 3 to + 3, [50]. This may lead to low sensitivity in detecting MCI and can be also due to the test’s lack of complexity as well as the absence of executive function items [51]. In addition, MMSE does not consider patient’s mood on the day when they take the test and it is possible that patients may be disrupted due to their current mental state, for example, depression or anxiety [52]. However, this has been controlled to the highest level as possible by controlling different co-variates affecting the MMSE scores including, depression scale and educational attainment. However, multiple MMSE tests over time can be used to predict the conversion of MCI to dementia [49], which was performed during the 10 years of data collection of KLoSA. While the subtype of the dementia is not identified in this study, limiting the association of the factors with a specific type, the factors associated with all-cause dementia are likely to be similar to AD with it being the majority of Korean dementia cases [32]. Moreover, the MMSE test provided an easy and quick administration, and is extensively used to distinguish between dementia and normal cognition.

## Conclusion

This study found that new diagnosis with CVDs (cerebrovascular disease and CHD) greatly increased the risks of developing dementia in a Korean population, while being diagnosed with CVDs before MCI had no effect on cognitive decline. This suggests that preventing patients with MCI from developing CVDs is crucial in delaying their cognitive decline rapidly advancing to dementia. Also, existing HBP and/or arthritis or rheumatism conditions increased the risks of developing cognitive decline leading to both MCI and dementia. In lieu of the protective factor of MCI and dementia, implementing regular exercise routine well before mid-life and cognitive decline is significant, with adjustments made for those suffering from health conditions, so they can continue exercising despite their morbidity. Finally, further attention in diabetes care and management is needed for patients who already show decline in cognitive ability, as it is likely that their MCI affect their ability to manage their existing chronic conditions and may adversely affect their cognitive ability further.

## Data Availability

The datasets generated during and/or analyzed during the current study are available from the corresponding author on reasonable request.

## Code availability

The codes generated during and/or analyzed during the current study are available from the corresponding author on reasonable request.
